# Machine learning approach to single nucleotide polymorphism-based asthma prediction

**DOI:** 10.1371/journal.pone.0225574

**Published:** 2019-12-04

**Authors:** Joverlyn Gaudillo, Jae Joseph Russell Rodriguez, Allen Nazareno, Lei Rigi Baltazar, Julianne Vilela, Rommel Bulalacao, Mario Domingo, Jason Albia

**Affiliations:** 1 Institute of Mathematical Sciences and Physics, University of the Philippines Los Baños, Philippines; 2 Genetics and Molecular Biology Division, Institute of Biological Sciences, University of the Philippines Los Baños, Philippines; 3 Philippine Genome Center Program for Agriculture, Office of the Vice Chancellor for Research and Extension, University of the Philippines Los Baños, Philippines; 4 Domingo Artificial Intelligence Research Center, Los Baños, Philippines; 5 Computational Interdisciplinary Research Laboratories (CINTERLabs), University of the Philippines Los Baños, Philippines; Instituto Nacional de Medicina Genomica, MEXICO

## Abstract

Machine learning (ML) is poised as a transformational approach uniquely positioned to discover the hidden biological interactions for better prediction and diagnosis of complex diseases. In this work, we integrated ML-based models for feature selection and classification to quantify the risk of individual susceptibility to asthma using single nucleotide polymorphism (SNP). Random forest (RF) and recursive feature elimination (RFE) algorithm were implemented to identify the SNPs with high implication to asthma. K-nearest neighbor (kNN) and support vector machine (SVM) algorithms were trained to classify the identified SNPs whether associated with non-asthmatic or asthmatic samples. Feature selection step showed that RF outperformed RFE and the feature importance score derived from RF was consistently high for a subset of SNPs, indicating the robustness of RF in selecting relevant features associated with asthma. Model comparison showed that the integration of RF-SVM obtained the highest model performance with an accuracy, precision, and sensitivity of 62.5%, 65.3%, and 69%, respectively, when compared to the baseline, RF-kNN, and an external MeanDiff-kNN models. Furthermore, results show that the occurrence of asthma can be predicted with an Area under the Curve (AUC) of 0.62 and 0.64 for RF-SVM and RF-kNN models, respectively. This study demonstrates the integration of ML models to augment traditional methods in predicting genetic predisposition to multifactorial diseases such as asthma.

## Introduction

Risk prediction of complex diseases such as asthma proves to be challenging due to the combinatorial effects of environmental and genetic factors. The major challenges are: 1) obtaining a sufficient sample size to develop univariate or multivariate predictor and, 2) equipping the predictor with biological variables associated with complex disease. In general, biological modelling studies for disease risk assessment using genetic information have been supplemented by several technologies and study designs. For example, genome-wide association studies (GWAS) use single-nucleotide polymorphism (SNP), a single-base pair change in the DNA sequence, as biomarkers to locate genetic regions associated with a particular trait. Recent GWAS [1–5] identified SNPs linked to candidate genes implicated to asthma. However, GWAS only accounts for a small fraction of individual traits, while heritability related to complex diseases remains unresolved, thus, the emergence of “mystery of missing heritability” phenomenon. Based on [6, 7], the estimated missing heritability of asthma from twin studies ranges from 35% to 70%.

One possible explanation for the “mystery of missing heritability” is the compounding biological interactions inherent to a complex disease [8]. Most of the biological modeling studies [9–11] which established SNP association with asthma still adopt the traditional statistical tests on individual SNP subject to a certain threshold, consequently neglecting the SNP-SNP interactions. Machine learning has the potential to unravel complex genetic and environment interactions constituting a disease by considering SNP-SNP interactions in model development. In genomic analysis, machine learning is employed to identify genetic variants and predict individual’s susceptibility to disease based on the identified biomarkers [12]. Despite the limited attempts, recent works have shown that machine learning-based approach augments the analysis on asthma at the genomic level, i.e., from genotype-phenotype association to phenotype prediction. For instance, Xu et al. [12] utilized random forest (RF) algorithm to identify relevant SNPs and to classify asthma case and control samples based on clinical data and SNPs.

Due to the well-known curse of dimensionality inherent to the SNP data, i.e., higher feature size compared to sample size, constructing accurate machine learning-based predictive models for complex disease presents a challenge [12]. One way to circumvent the curse of dimensionality problem is to integrate various machine learning algorithms. The general approach is to employ feature selection method on the SNP data before classifying samples using machine learning-based classifiers. For example, Mieth et al. [13] used support vector machine (SVM) to select relevant SNPs associated with breast cancer. SVM along with Naive Bayes and decision trees have also been used to identify breast cancer cases using SNPs selected via information gain [14]. Furthermore, mean difference calculation and k-nearest neighbor (kNN) have also been employed to quantify SNP relevance and to perform classification task on the breast cancer dataset [15], respectively.

In this work, we developed a novel machine learning-based approach to quantify the individual’s susceptibility to asthma based on SNP data. We implemented a two-step approach which involves feature selection and classification steps. The RF and RFE were utilized to select SNPs highly associated to asthma, while SVM and kNN were employed to quantify the propensity of an individual to develop asthma. The predictive models were evaluated using the following metrics: accuracy, sensitivity, precision, and area under the curve (AUC). In the next section, we describe the procedure of data collection, preprocessing, SNP selection, and classification. Finally, the results of integrating feature selection and classification methods will be compared with baseline and external models.

## Materials and methods

### Data collection and preprocessing

The SNP data was obtained from openSNP database [16], a public repository from 23andMe, deCODEme, and FamilyTreeDNA. The raw dataset is composed of an MS Excel file containing phenotypes information of users, and text files containing SNP data encoded in Variant Call Format (VCF). Each text file contains meta-information lines and a header line with the following field names: SNP ID, chromosome number, position, and genotype. Phenotypes irrelevant to the study were eliminated and users with incomplete report on asthma were discarded. The cleaned data is composed of 143 samples which represents the users with complete asthma report, 1,088,991 SNPs as features of each sample, and class column that labels each sample based on their phenotype (non-asthmatic or asthmatic).

We adopted similar quality control procedure from [15] which involves missing call filtering and deviation from Hardy-Weinberg equilibrium to select the SNPs. For the missing call filtering, we formulated criteria based on the quality and characteristic of the collected data. The first criterion excludes SNPs whose percentage of present samples is ≤ 98%. For the second criterion, the samples were ranked based on the percentage of present SNP in which 90% of the samples were retained. The Hardy-Weinberg equilibrium principle states that in an infinitely sized population with no selection, migration and mutation, random mating will bring about constant allele and genotype frequencies across generations. Genotypes at a bi-allelic locus can then be predicted to have the following distribution of frequencies:

$${\text{p}}^{2}+2\text{pq}+{\text{q}}^{2}=1$$ (1)

where *p*^2^ is the frequency of homozygotes for allele *p*, 2*pq* is the frequency of heterozygotes for alleles *p* and *q*, and *q*^2^ is the frequency of homozygotes for allele *q*. SNPs that deviated from Hardy-Weinberg equilibrium due to selection, population admixture, cryptic relatedness, genotyping error and genuine genetic association [17] were excluded in the dataset. Chi-square test was implemented using Hardy-Weinberg package in R [18] on allele frequencies of each SNP from the control samples, wherein SNPs with *p* − *value* < 0.001 were discarded. After data preprocessing and quality control, 128 samples (57 samples with asthma condition during childhood and adulthood and 71 control samples are unaffected with asthma) were retained. A total of 176,288 SNPs was retained with genotypes comprised of minor and major alleles. Finally, genotypic encoding was implemented wherein homozygous, heterozygous, and variant homozygous SNPs were represented by 1, 2, 3, respectively.

### Feature selection

Disease detection driven by high-dimensional data entails the use of feature selection method to identify the features highly associated to the phenotype [19]. In this study, RF algorithm which involves building multiple decision trees based on relevant SNPs was used. RF outperforms other feature selection methods in terms of robustness as a result of its ensemble trait of measuring the predictive importance of each feature to build efficient decision trees [19]. The RF algorithm was trained using the following input matrix,

$${\text{D}}_{\text{N}}=\left\{\;(\;X_{1},Y_{1}),\;(\;X_{2},Y_{2}),\;\ldots ,(\;X_{N},Y_{N})\right\}$$ (2)

where *N* is the number of samples, and *X*_*i*_ and *Y*_*i*_ are the SNP values and the output class of *i*^*th*^ sample, respectively. For each data point, an out-of-bag error (OOBE) and the mean over the forest were calculated. After training, the *j*^*th*^ SNP was permuted among the training data and the difference the OOBE before and after permutation was calculated. To obtain the importance score of the *j*^*th*^ SNP, the mean of the differences were calculated and normalized.

### Model construction

To predict the individual’s susceptibility to asthma, two different integrated machine learning models were constructed. These models are RF-kNN and RF-SVM which both employed RF as the feature selection algorithm to identify the relevant SNPs. Subsequently, the selected SNPs were used as inputs to kNN and SVM classifiers.

#### k-Nearest Neighbor (*kNN*)

kNN algorithm is a nonparametric lazy learner due to the absence of a training phase when performing classification tasks. In this work, the kNN algorithm was implemented on the SNP data to construct a predictive model that classifies non-asthmatic and asthmatic samples. A particular set of SNPs associated to a sample is represented as a continuous variable that can be plotted into the feature space. Hence, given a sample with SNP as features and an unknown label, the kNN algorithm gives prediction by performing a majority voting on the output class of the sample’s *k* nearest neighbors. For further discussion of kNN using SNP data, refer to [15].

To implement kNN, we define *X*_*i*_ be the *p*-dimensional input vector of the *i*^*th*^ sample, and is defined as,

$$\begin{array}{c} {\text{X}}_{\text{i}}=\;(\;SNP_{i1},\;SNP_{i2},\;\ldots ,SNP_{ip}),i=\;1,\;2,\;\ldots ,p \end{array}$$ (3)

where *p* is the total number of SNPs. The corresponding output vector *Y*_*i*_ is given by,

$${\text{Y}}_{\text{i}}\in \;\left\{\;0,1\right\},i=\;1,\;2,\;\ldots ,N$$ (4)

where *N* is the total number of samples. The dataset are plotted on a multi-dimensional feature space with their corresponding class label. To determine the class of an unseen sample *U*, the *k* nearest neighbor is determined by calculating the Euclidean distance *d*(*U*, *X*_*i*_) relative to the other labeled samples. The Euclidean distance between *U* and *X*_*i*_ is given by,

$$\text{d}(\;\text{U},{\text{X}}_{\text{i}})=\sqrt{\sum_{j=1}^{p}{\left(U_{j}-X_{ij}\right)}^{2}}$$ (5)

where *j* represents a certain SNP. We note that our selection of Euclidean distance as a similarity measure is motivated by numerous literatures [14, 15, 20] which extensively used this metric to analyze SNPs. Furthermore, to make our work more comprehensive, we tested several distance metrics and compared their performance with the Euclidean distance (see S2 Table).

#### Support vector machine (*SVM*)

SVM is a learning algorithm used in tasks such as non-linear classification [11, 13, 14], function estimation, and density estimation. The learning algorithm constructs a hyperplane that provides maximum separation or margin, between classes. For detailed discussion of SVM implementation on SNP data, refer to [13, 14].

The construction of the classification model can be viewed as an optimization problem. To implement SVM, the training vector representing SNP values and its corresponding label is given by,

$${\text{D}}_{\text{i}}={\left\{(\;X_{i},Y_{i})\right\}}_{i=1}^{N}$$ (6)

where *N* is the total number of samples, and *X*_*i*_ and *Y*_*i*_ represent the SNP values and class label of the *i*^*th*^ sample, respectively. The algorithm minimizes,

$$\frac{1}{2}{∥w∥}^{2}+C\sum_{i=1}^{n}\varepsilon_{i}$$ (7)

subject to the following condition,

$$Y_{i}(w^{T}X_{i}+b)\geq \;1-\varepsilon_{i},\varepsilon_{i}\geq 0,\forall_{i}$$ (8)

where *w* is learning weight vector, b is the bias, *ε* is the error term, and *C* is the tradeoff parameter between the error and margin. After finding the optimal solution, the function,

$$\text{f}(\text{z})=sgn(w^{T}z+b)$$ (9)

is used to predict the class of a unseen sample. The sign of the function is given by,

f(z)∈{-1,+1} (10)

wherein positive (+) and (-) values indicate non-asthmatic and asthmatic, respectively.

### Hyperparameter selection and performance evaluation

One crucial step in constructing a machine learning predictive model is the selection of hyperparameters to achieve the highest model performance. Shown in Table 1 are the tested hyperparameter and their corresponding test values to train and optimize the machine learning algorithms.

**Table 1:** List of hyperparameter values.

Method	Hyperparameter	Range of values
Random Forest	optimum number of trees in the forest, n_estimators	{20, 30, 40, 50, 60, 70, 80, 90, 100, 110, 120, 130, 140, 150, 160, 170, 180, 190, 200}
	maximum number of features considered for splitting a node to achieve least uncertainty when creating a tree, max_features	{400, 500, 600}
SVM	kernel	{linear, sigmoid, rbf, poly}
	regularization parameter, C	{1, 10, 100, 1000}
	tolerance, *ε*	{0.002, 0.1, 0.001, 0.0001}
kNN	number of neighbors, k	1, 3, 5, 7, 9, 11, 13, 15, 17, 19, 21, 23, 25, 27, 29, 31, 33, 35

Cross-validation techniques were employed to estimate the model performance on the unseen data. The RF classifier was evaluated using stratified k-fold cross validation (*k* = 10) which ensures that each fold contains roughly the same number of samples per class. RF-SVM and RF-kNN were evaluated using leave-one-out cross-validation (LOOCV), which discards a single sample to be used as a test set before training the model on the remaining samples; hence, k is equal to the number of samples (*k* = 128). The performance metrics used to evaluate the models were accuracy (ratio of correctly predicted observations to the total observations), precision (ratio of correctly predicted positive observations to the total predicted positive observations), and sensitivity (ratio of correctly predicted positive observations to the actual positive observations). The AUC—receiver operating characteristic (ROC) curve is another measure of performance of a machine learning classifier model. The ROC is a probability curve while the AUC represents the capability of the model to distinguish among classes. The ROC curve is constructed by plotting the true positive rate versus false positive rate.

To further evaluate the results, we compare the integrated models with baseline model and an external model [15] implemented for breast cancer prediction. Scripts were developed using Python programming language and simulations were executed in a machine with 2.3 GHz Intel Core i5 processor, 8Gb random access memory (RAM), and 2 cores.

## Results and discussion

### Feature selection

**Fig 1:**
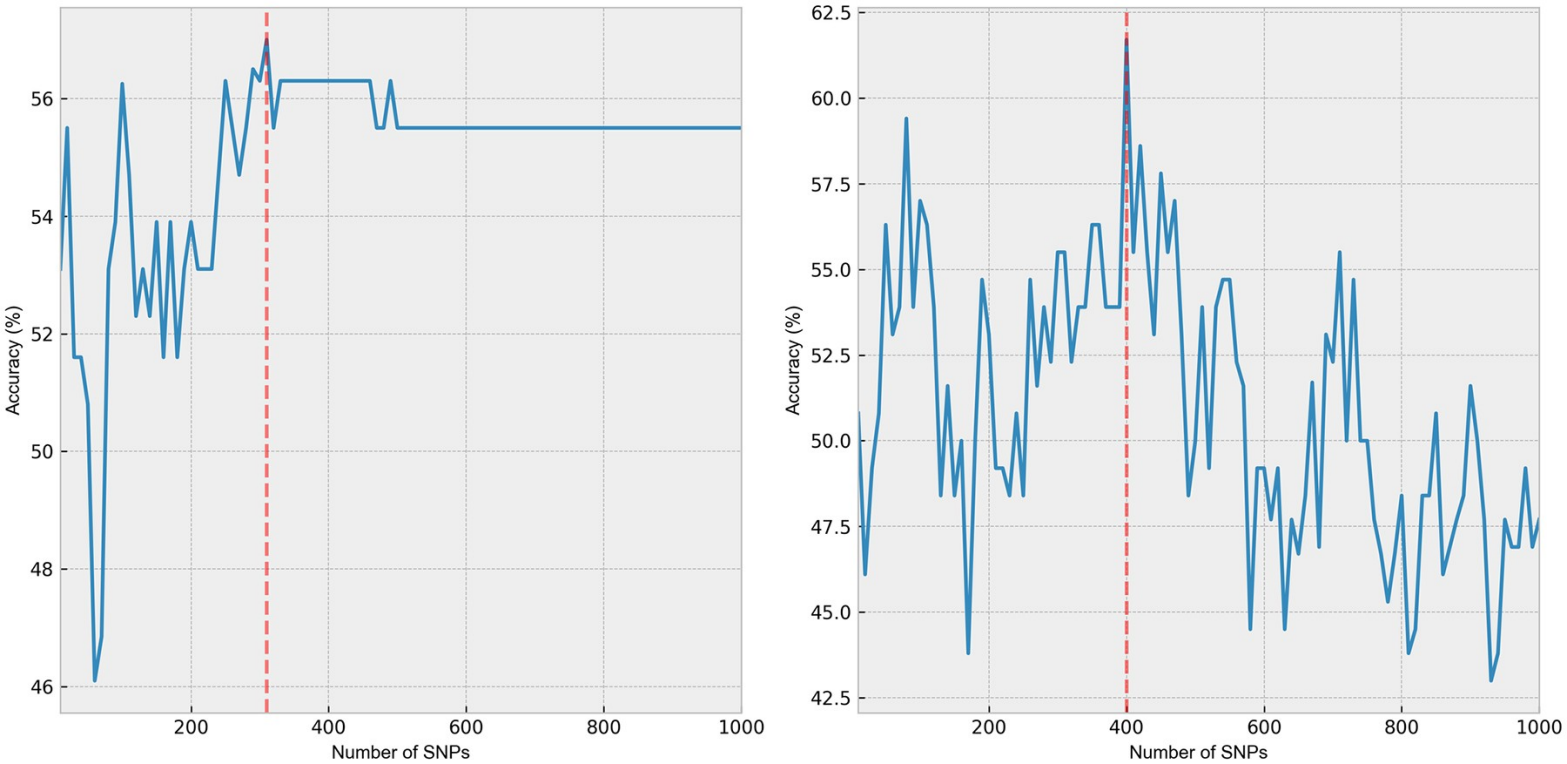
Accuracy vs. number of SNP for SVM (left) and kNN (right).

We optimized the hyperparameters to obtain the best RF architecture. Results show that the highest performance having accuracy, precision, and sensitivity of 68.75%, 83.3%, and 68%, respectively, is obtained when *n_estimators* = *40* and *max_features* = *400*. This optimum RF architecture is then used to select the SNPs which will serve as input to train the SVM and kNN classifiers. Training phase revealed that the highest accuracy for RF-SVM and RF-kNN were obtained when the optimum number of features are 310 and 400, respectively, see Fig 1.

To further evaluate the performance of RF in selecting highly associated SNPs, we implemented recursive feature elimination (RFE) in combination with SVM and kNN. As a feature selection method, RFE initially builds a model on the entire dataset and computes feature importance score for each predictor. For the next iteration, the predictor with the lowest feature importance score is omitted from the dataset that will be used to rebuild the model. Result shows (see Table 2) that RF when integrated to SVM and kNN classifiers has a better performance as a feature selection method compared with RFE.

**Table 2:** Comparison of feature selection methods.

Model	Accuracy (%)
RF-SVM	62.17
RF-kNN	61.70
RFE-SVM	50.90
RFE-kNN	49.68

**Fig 2:**
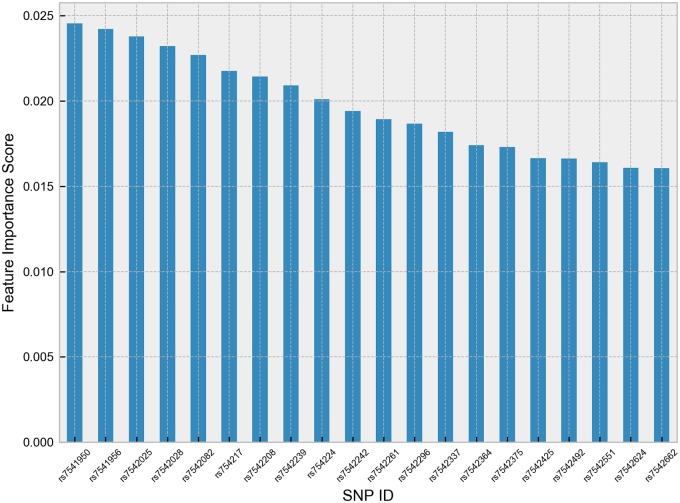
Feature importance score of top 20 SNPs.

Shown in Fig 2 is the feature importance scores of top 20 SNPs. Table 3 shows the characteristics of the top five SNPs that were selected by the RF based on feature importance score. Based on the feature importance score, the top five SNPs were consistently selected by RF in every fold during the cross-validation procedure. This result is indicative that integration of RF with classifiers is robust in selecting important SNPs. Favoring a robust feature selection algorithm apart from classification performance is highly desirable to further gain insight on the SNP data. Moreover, the robustness of the RF can be attributed to its stability in selecting important features, i.e., RF is insensitive to perturbations in the dataset [19].

**Table 3:** Characteristics of top five SNPs selected by RF based on importance score [21].

SNP ID	Chromosome Location	Gene	Functional Consequence	GeneCard Summary
rs7541950	1:147903855	*GJA8*	intron variant	GJA8 (Gap Junction Protein Alpha 8) is a Protein Coding gene. Diseases associated with GJA8 include Cataract 1, Multiple Types and Cataract Microcornea Syndrome. Among its related pathways are Development Slit-Robo signaling and Vesicle-mediated transport. Gene Ontology (GO) annotations related to this gene include channel activity and gap junction channel activity
rs7541956	1:111366426	*LOC105378904*	intron variant	RNA Gene, and is affiliated with the ncRNA class
rs7542025	1:40643890	*RIMS3*	intron variant	RIMS3 (Regulating Synaptic Membrane Exocytosis 3) is a Protein Coding gene. Gene Ontology (GO) annotations related to this gene include ion channel binding
rs7542028	1:168718584	*DPT*	intron variant	DPT (Dermatopontin) is a Protein Coding gene. Diseases associated with DPT include Commensal Bacterial Infectious Disease and Anisometropia
rs7542082	1:118617643	NA	NA	NA

While the asthma-associated SNPs selected by RF has low feature importance scores, it is interesting to note that prior studies have not identified these SNPs to be associated to asthma, nor included in the 19 asthma-associated SNPs [22] present in the dataset. This observation is attributed to: 1) various sequencing platforms were used which may lead to the exclusion of lung-function associated SNPs or affect the phenotypic variance within the population; 2) as demonstrated in [23–26], RF takes into account SNP-SNP interactions while the traditional approach only performs direct genotype-phenotype association testing. Considering the result of our feature selection scheme, in which the selected SNPs are currently excluded in the list of known SNPs associated to asthma [22], we posit that the integration method can be utilized as a preliminary method to discover new biomarkers associated with complex diseases.

### Model comparison

The SVM and kNN were implemented on both entire and selected dataset to establish baseline models. Furthermore, a more stringent test on the performance of SVM and kNN classifiers were implemented on the 19 asthma-associated SNPs. Considering the higher accuracy of RF as a feature selection method when integrated to SVM and kNN as discussed above, we compare the performance of RF-SVM and RF-kNN with the baseline model. Table 4 shows that the integrated models obtained higher classification performance than the baseline models.

**Table 4:** Comparison of performance between RF-SVM and RF-kNN to the baseline SVM and kNN models. The selected SNPs for Baseline SVM and kNN refers to the 19 asthma-associated SNPs.

Models	Accuracy (%)	
	Entire Dataset	Selected SNPs
Baseline SVM	52.83	51.81
Baseline kNN	49.69	49.09
RF-SVM	56.30	62.17
Baseline kNN	54.70	61.70

**Fig 3:**
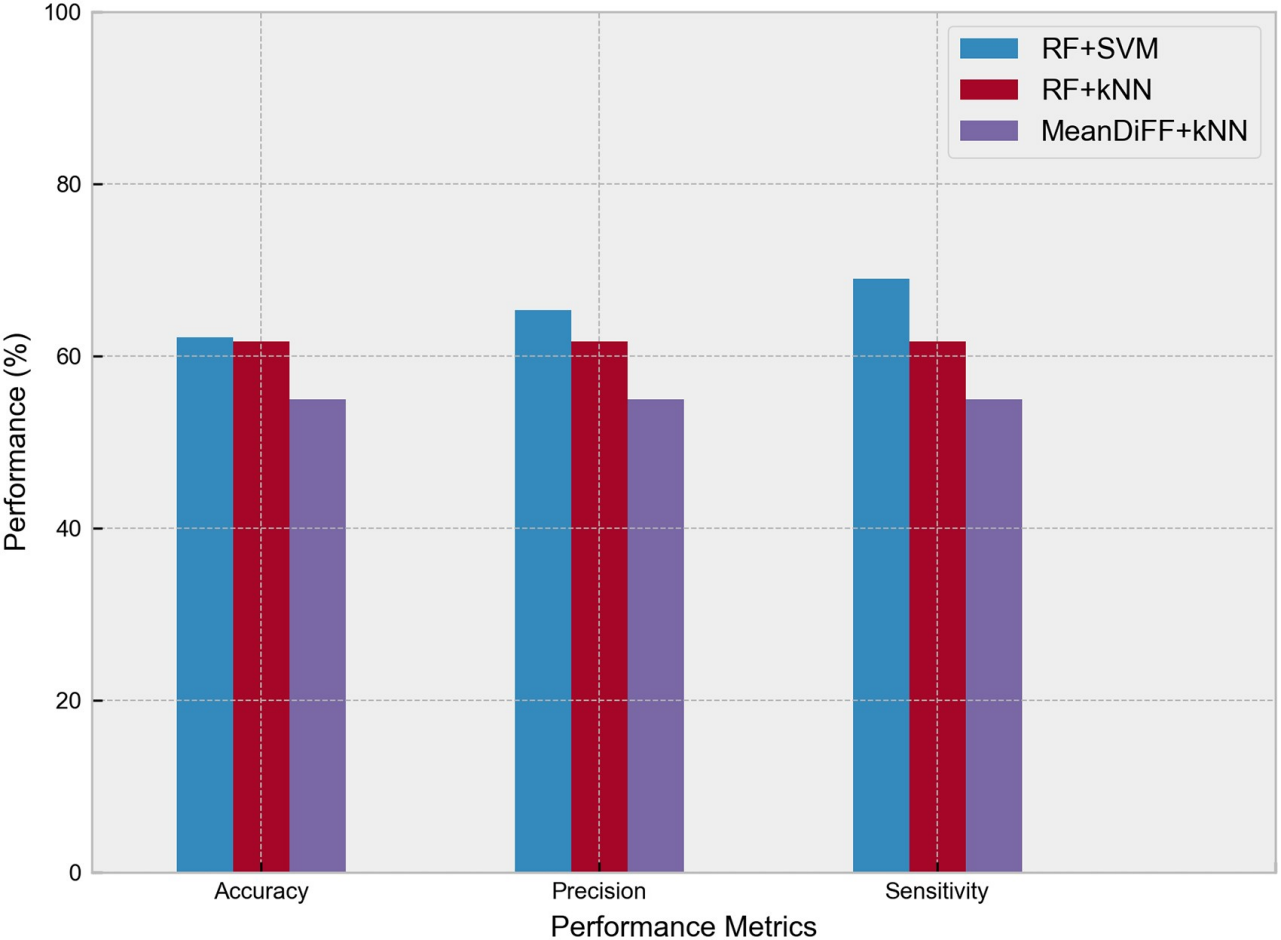
Performance of the three ML models (RF-SVM, RF-kNN, MeanDiff-kNN).

To further evaluate the performance of the integrated models, MeanDiff-kNN derived from [15] was implemented on the dataset. Fig 3 shows the comparison of the three integrated models RF-SVM, RF-kNN, MeanDiff-kNN. The RF-SVM attained the highest performance among the models with an accuracy, precision, and sensitivity of 62.17%, 65.3%, and 69%, respectively. We note that the baseline model [15] previously proposed for breast cancer detection when applied to the asthma dataset achieved 55% accuracy. Similar performance metrics percentage of 61.7% and 55% were attained for RF-kNN and MeanDiff-kNN, respectively. The SVM outperformed other classifiers in predicting asthma susceptibility by considering the interactions among SNPs to construct a more suitable predictive model tailored to the input data [13, 14, 27]. Compared to other machine learning methods, SVM is very powerful in detecting intricate trends and patterns in complex genomic dataset [20]. While SVM has better performance in high-dimensional data with few samples, kNN works well with data points in low-dimensional space. Shown in Table 5 is the optimal hyperparameters for each model.

**Table 5:** Optimal hyperparameters determined for the models.

Method	Hyperparameter values
SVM	kernel = rbf
	regularization parameter, C = 100
	tolerance, *ε* = 0.001
	number of selected SNP = 310
kNN	number of nearest neighbors, k = 7
	number of selected SNP = 400

**Fig 4:**
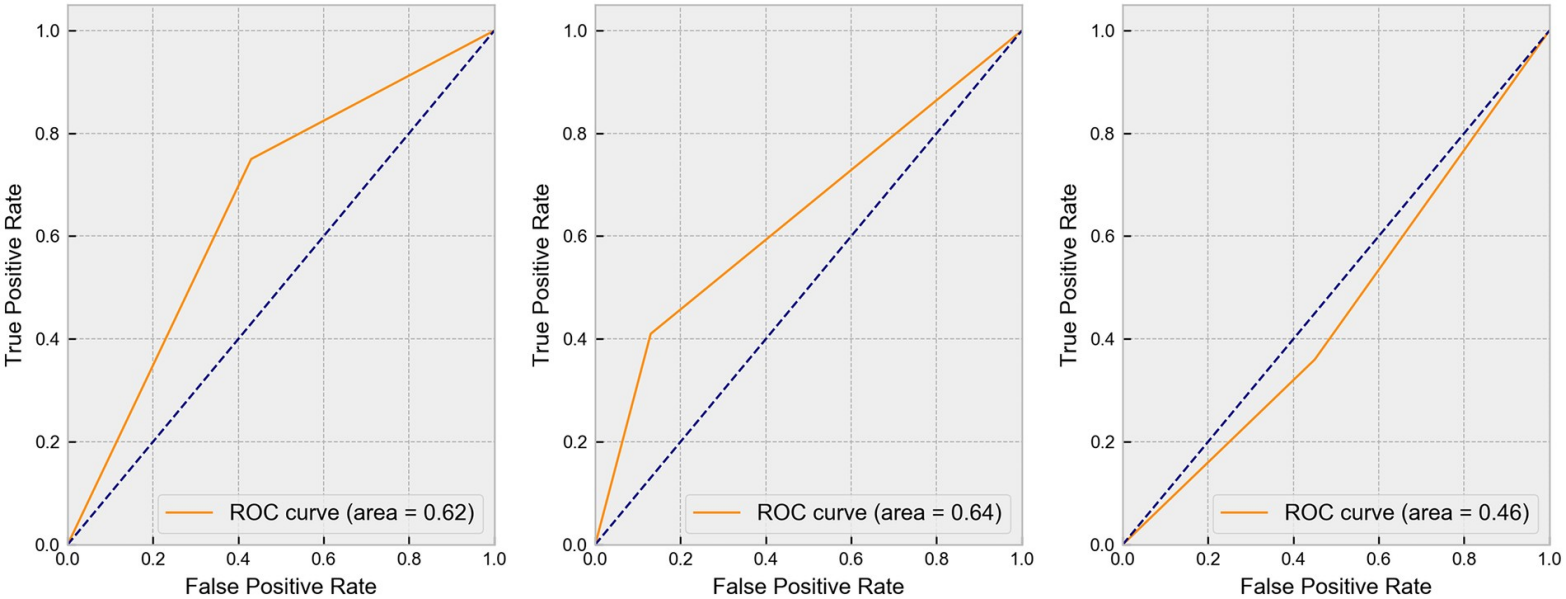
ROC Curves of RF-SVM (a), RF-kNN (b), and MeanDiff-kNN (c).

Fig 4 shows the ROC curves of RF-SVM, RF-kNN, and MeanDiff-kNN. Among the integrated models, RF-kNN attained the highest AUC of 0.64. The AUC of the RF-SVM and RF-kNN were comparable to the results in [12] which reported an AUC of 0.66 using 160 SNPs and clinical data to predict severe asthma exacerbations. Despite the absence of clinical data in the predictor variables, our integrated models obtained good performance in distinguishing case from control samples. Moreover, the integrated models achieved good prediction results, notwithstanding the limited sample size. This further indicate that the combination of machine learning algorithms used in the proposed models are able to predict asthma susceptibility with satisfactory performance despite the limited biological variables used in the model construction.

## Conclusion

We developed machine learning-based predictive models which utilized SNP data to quantify the individual susceptibility to asthma. More precisely, we employed an integrated approach consisting of a feature selection step to identify the SNPs highly associated to asthma, and a classification step to predict asthma susceptibility. Results show that the integrated RF-SVM model achieves the highest accuracy, precision, sensitivity, and AUC compared to RF-kNN and baseline models. Furthermore, this study demonstrated that the integration of various machine learning methods is a well-suited approach to investigate high dimensional SNP data for genotype-phenotype association and phenotype prediction. To a large extent, we have shown how machine learning approach can augment the insights derived from GWAS in analyzing massive and complex biological data obtained from next generation sequencing (NGS) platforms. The integrated approach can be naturally extended on a wide variety of biological data such genetic mutations, copy number variations as well as clinical data which may take into account environmental factors to come up with a more holistic understanding of genetic etiology of complex diseases.

## Source code

The Python codes as well as the SNP data used in this study is available in this github repository.

## Supporting information

S1 Table Feature importance table. (PDF)

S2 Table Distance metrics performance table. (PDF)
