# Supplementary material for: Machine learning approach to single nucleotide polymorphism-based asthma prediction
Source: PLoS One. 2019 Dec 4;14(12):e0225574. doi: 10.1371/journal.pone.0225574 (PMC6892549; doi:10.1371/journal.pone.0225574)
Supplement: S1 Table — (PDF) [file pone.0225574.s001.pdf]

Supporting Information

S1 Table: Feature Importance Table

SNP	Feature Importance Score	SNP	Feature Importance Score
rs7541950	0.024542574	rs7543182	0.010722406
rs7541956	0.024221491	rs7543277	0.010612024
rs7542025	0.023796671	rs754335	0.010416667
rs7542028	0.02322492	rs7543364	0.010339463
rs7541950	0.024542574	rs7543435	0.010078105
rs7541956	0.024221491	rs754344	0.00989011
rs7542025	0.023796671	rs7543462	0.009754704
rs7542028	0.02322492	rs7543481	0.009722979
rs7542082	0.022711435	rs7543486	0.009596641
rs754217	0.021761956	rs7543509	0.009230769
rs7542208	0.021429178	rs7543524	0.008514412
rs7542239	0.020914363	rs7543621	0.008391608
rs754224	0.020108542	rs7543626	0.008054245
rs7542242	0.019414212	rs754363	0.007680117
rs7542261	0.018938898	rs7543680	0.00756925
rs7542296	0.018679718	rs7543683	0.007393256
rs7542337	0.018196489	rs7543711	0.007078934
rs7542364	0.017405561	rs7543730	0.007036189
rs7542375	0.017296894	rs7543757	0.006815796
rs7542425	0.016658331	rs7543788	0.006666667
rs7542492	0.016629712	rs7543847	0.006318561
rs7542551	0.016418684	rs754390	0.006169269
rs7542624	0.0160841	rs754394	0.005169732
rs7542662	0.016060108	rs754396	0.004195103
rs7542665	0.016057776	rs754410	0.004191474
rs7542735	0.016040554	rs7544118	0.004169884
rs7542768	0.015814954	rs754413	0.004166164
rs7542797	0.015616558	rs7544201	0.004152393
rs7542810	0.015111798	rs7544329	0.004136604
rs7542860	0.014918073	rs7544348	0.004078572
rs7542878	0.014621085	rs7544357	0.004075924
rs7542884	0.014072377	rs7544358	0.00406446
rs7542900	0.013637332	rs754437	0.004059829
rs754291	0.013233819	rs7544426	0.004050359
rs754292	0.012863495	rs7544479	0.004048769
rs7543038	0.012771619	rs7544500	0.004032258
rs7543046	0.012587413	rs7544557	0.004030589
rs7543048	0.011879632	rs7544572	0.004015686
rs7543057	0.011825573	rs7544587	0.003956044
rs7543061	0.01125	rs7544608	0.003952755
rs7543063	0.011239024	rs7544611	0.003951961
rs7543064	0.011195471	rs7544659	0.003856838
rs754313	0.010994045	rs7544736	0.003848004
rs7543130	0.010940638	rs7544745	0.003819444
rs7543144	0.010878191	rs7544775	0.003780242
rs7543148	0.010870076	rs7544813	0.003685827
