# Supplementary material for: Machine learning approach to single nucleotide polymorphism-based asthma prediction
Source: PLoS One. 2019 Dec 4;14(12):e0225574. doi: 10.1371/journal.pone.0225574 (PMC6892549; doi:10.1371/journal.pone.0225574)
Supplement: S2 Table — (PDF) [file pone.0225574.s002.pdf]

Supporting Information

S2 Table: Distance Metrics Performance Table

Distance Metrics	Accuracy (%)
Euclidean	61.7
Manhattan	49.06
Cosine	48.28
Cityblock	49.06
Jaccard	45.91
Hamming	50.31
Chebyshev	41.5
Braycurtis	47.8
Correlation	49.05
Canberra	49.69
Dice	45.91
Kulsinski	46.54
